# The Effect of Particle Shape on Sintering Behavior and Compressive Strength of Porous Alumina

**DOI:** 10.3390/ma11071137

**Published:** 2018-07-04

**Authors:** Kimiya Miyake, Yoshihiro Hirata, Taro Shimonosono, Soichiro Sameshima

**Affiliations:** Department of Chemistry, Biotechnology, and Chemical Engineering, Kagoshima University, Kagoshima 890-0065, Japan; k7050973@kadai.jp (K.M.); shimonosono@cen.kagoshima-u.ac.jp (T.S.); samesima@cen.kagoshima-u.ac.jp (S.S.)

**Keywords:** particle shape, porous alumina, sintering, Young’s modulus, grain boundary, compressive strength

## Abstract

Alumina particles with different shapes, such as sphere, rod, and disk, were examined for the sintering behavior and compressive strength of partially sintered porous alumina. While both the spherical and disk-like particles were packed well to the relative density of 61.2–62.3%, the packing density of rod-like particles was only 33.5%. The sintering rate of alumina particles increased in the order of disk < rod < sphere. The compressive strength of sintered porous alumina was higher for the spherical particles than for the rod-like and disk-like particles. The uniform distribution of the applied load over many developed grain boundaries contributed to the increase in the compressive strength for the spherical particles. The applied load concentrated on a few grain boundaries of rod-like or disk-like particles, caused fracture at a low compressive stress.

## 1. Introduction

Porous ceramic materials have been used in the fields of thermal insulators [1,2], adsorbents [3,4], catalyst supports [5,6], electrodes in solid oxide fuel cells [7,8], or filtration membranes [9,10]. Its pore structure significantly affects the mechanical properties and the strength of porous ceramics decreases exponentially with increasing porosity [11]. Rice studied the correlation of pore shape and stress concentration on a minimum solid area in the elastic deformation of ceramics [12]. Ostrowski et al. [13] report that the Young’s modulus of porous ceramics increases with the contact area between adjacent grains and is expressed by Equation (1),

(1) $$E_{\mathrm{porous}}{=E}_{\mathrm{dense}}{\left(1-\frac{P}{P_{\mathrm{green}}}\right)}^{n}$$

where *E_porous_* is the Young’s modulus of partially sintered porous ceramics with porosity (*P*), *E_dense_* is the Young’s modulus for fully dense ceramics, and *P_green_* is the porosity of the green body before sintering. The exponent *n* is fitted to be 1.15 in Reference [2] and *E_porous_* approaches *E_dense_* at *P* = 0%. Kim et al. reported the compressive strength of microcellular mullite where small spherical cells (≤ 20 μm) were distributed homogeneously. The controlled cell size and porosity are key factors to fabricate a strong porous structure [14]. In our previous papers [15,16,17,18], we proposed Equation (2) for the compressive strength (*σ_p_*) of partially sintered porous alumina,

(2) $$\sigma_{p}{=\sigma }_{0}{\pi y}^{2}{\left(\frac{N}{V}\right)}^{2/3}$$

where *y* is the radius of a circular grain boundary and *N* is the number of spherical grains in the bulk volume (*V*) of a sintered porous compact. The *σ*_0_ value corresponds to the strength needed to fracture the several grain boundaries (*n*) surrounding three-dimensionally one grain at 0% porosity, and is expressed by Equation (3),

(3) $$\sigma_{0}=\frac{1}{2}n\;\sigma (\mathrm{dense})$$

where *n* is the coordination number of grains and *σ*(dense) is the compressive strength at 0% porosity. The grain boundary area (*A_gb_*) for one grain layer per unit area (1 m^2^) of a porous ceramics perpendicular to the compressive direction is given by Equation (4) from Equations (2) and (3).

(4) $$A_{\mathrm{gb}}=\frac{1}{2}{n\pi \;y}^{2}{\left(\frac{N}{V}\right)}^{2/3}$$

Equation (2) indicates that the increase in the grain boundary radius and the grain number leads to the increase in the compressive strength. The development of grain boundaries with sintering is related to the decrease in the specific surface area of the porous ceramic compact [15,16]. The area (*A_gs_*) of the gas–solid interface and the grain boundary area (*A_pp_*) of the particle–particle interface per particle of radius *r* are expressed by Equations (5) and (6), respectively,

(5) $$A_{\mathrm{gs}}{=4\pi r}^{2}{-\mathrm{nS}}_{1}{=4}^{2/3}{\pi r}_{0}^{2}\frac{4-2\mathrm{np}}{{\left({4-3\mathrm{np}}^{2}{+\mathrm{np}}^{3}\right)}^{2/3}}$$

(6) $$A_{\mathrm{pp}}=\frac{1}{2}{n\pi y}^{2}{=2}^{1/3}{\pi r}_{0}^{2}n\frac{{2p-p}^{2}}{{\left({4-3\mathrm{np}}^{2}{+\mathrm{np}}^{3}\right)}^{2/3}}$$

where *S*_1_ is the disappeared surface area due to sintering, *r*_0_ is the radius of the starting particles and *p* is the ratio of the shortened distance (*h*) between two particles to the particle size (*r*) (*p* = *h*/*r*) during sintering. The *p* value can be determined from a specific surface area by assuming no change in the particle number in one sintered powder compact using Equation (7) [16].

(7) $$\frac{S}{S_{0}}=\frac{A_{\mathrm{gs}}}{A_{0}}=\frac{4-2\mathrm{np}}{4^{1/3}{\left({4-3\mathrm{np}}^{2}{+\mathrm{np}}^{3}\right)}^{2/3}}$$

*S*_0_ represents the specific surface area before sintering and *S* represents the specific surface area after sintering, *A*_0_ is equal to 4*πr*_0_^2^, and *p* is obtained from the ratio of the specific surface area before and after sintering. Another way to determine the *p* value is to apply Equations (8) and (9) regarding the relative density *D* and the linear shrinkage *q*, respectively, for *p* value [15,18],

(8) $$D={\left(\frac{1}{1-q}\right)}^{3}D_{0}$$

(9) $$q=\frac{h}{r_{0}}=p{\left(\frac{4}{{4-3\mathrm{np}}^{2}{+\mathrm{np}}^{3}}\right)}^{1/3}$$

where *D*_0_ represents the relative density before sintering. The relative density (*D*) of sintered porous compact is approximated by Equation (10) [18].

(10) $$D=\frac{\frac{4}{3}{\pi r}_{0}^{3}N}{V}$$

The *N*/*V* value (=3*D*/4*πr*_0_^3^) in Equation (10) is connected to the (*N*/*V*)^2/3^ value in Equation (2), giving the relation of (*N*/*V*)^2/3^ ≈ *D*^2/3^/(2.599*r*_0_^2^). This relation is substituted for Equation (2) to give Equation (11).

(11) $$\sigma_{p}={1.209\sigma }_{0}{\left(\frac{y}{r_{0}}\right)}^{2}D^{2/3}$$

Equation (11) suggests that the increase in the grain boundary area and relative density dominates the compressive strength of a sintered porous material. In our previous papers [15,18], (*y*/*r*_0_)^2^ value is expressed by Equation (12) as a function of shrinkage *p*.

(12) $${\left(\frac{y}{r_{0}}\right)}^{2}{=2}^{4/3}\frac{\left({2p-p}^{2}\right)}{{\left({4-3\mathrm{np}}^{2}{+\mathrm{np}}^{3}\right)}^{2/3}}$$

The compressive strength of the sintered porous compact is easily predicted from Equations (11) and (12), and expressed by Equation (13) for a constant *n* value.

(13) $$\sigma_{p}(\mathrm{fracture})={3.046\sigma }_{0}\frac{\left({2p-p}^{2}\right)}{{\left({4-3\mathrm{np}}^{2}{+\mathrm{np}}^{3}\right)}^{2/3}}D^{2/3}$$

The above analyses are derived for spherical grains. The purpose of this paper is to clarify the influence of particle shape on the sintering behavior and the compressive strength of partially sintered ceramics. It is reported that the *tert*-butyl alcohol-based gel-casting method provides a porous microstructure of inter-locked hexagonal plate-like alumina grains [19]. This structure exhibited a high porosity of 66.4% and a low compressive strength of 4.80 MPa. According to Faber and Evans [20], the mixing of needle-like grains with a high aspect ratio in the dense microstructure of equi-axised grains increases the fracture toughness. Similarly, whisker-reinforced composites have been widely studied to increase the mechanical properties of brittle ceramic materials. The addition of 10 mass% Al_2_O_3_ whiskers with 100–200 nm length and 20 nm width to matrix WC particles enhanced the flexural strength as compared with the powder composite of the 10 mass% Al_2_O_3_–90 mass% WC system [21]. The strengthening mechanisms were attributed to crack deflection, crack bridging, and ligamentary bridging between crack surfaces. However, few papers have reported the mechanical properties of porous ceramics prepared from whiskers or rod-like particles. In this paper, the sintering behavior and the compressive strength were examined for the alumina porous compacts prepared from spherical, rod-like and disk-like alumina particles. The goal of this paper is to identify the key factors affecting the mechanical properties of the partially sintered porous ceramics by comparing the measured strength and the proposed theoretical analysis. It is clearly demonstrated that the compressive strength, which is greatly affected by the shape of starting particles, depends on the number (*n_f_*) of grain boundaries which originally surround one grain and are fractured by a compressive stress. The uniform distribution of the applied load over many grain boundaries is a key factor to increase the compressive strength of partially sintered porous ceramics.

## 2. Experimental Procedure

### 2.1. Analysis of Starting Alumina Powders

**Figure 1:**
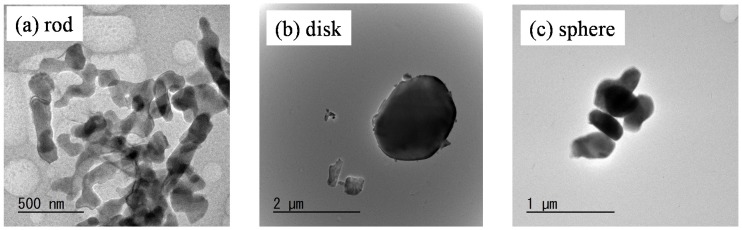
Particle morphology of (**a**) rod-like alumina, (**b**) disk-like alumina and (**c**) spherical alumina.

Table 1 shows the characteristics of the following three kind of α-alumina powders with different particle shape: (a) rod-like particles, impurity Na: 20 ppm, Si: 5 ppm, Fe: 8 ppm; (b) disk-like particles, chemical composition (mass %) Al_2_O_3_: 99.8, SiO_2_: 0.03, FeO_2_: 0.02, Na_2_O: 0.02, Ig. loss: 0.12; (c) spherical particles, Al_2_O_3_ purity > 99.99 mass%. The phases of alumina powders were identified by X-ray diffraction analysis (RINT 2200PCH/KG, Rigaku Co. Ltd., Tokyo, Japan). Particle morphology of alumina was observed by transmission electron microscope (TEM, JEM-3010, JEOL Ltd., Tokyo, Japan) and field emission electron microscope (S-4100H FE-SEM, Hitachi High-Tech Technologies Co., Tokyo, Japan). The dilute aqueous suspensions of the alumina particles at pH 3 were prepared and dropped on collodion membranes for TEM observation. The specific surface areas of alumina powders were measured by the Brunauer–Emmett–Teller (BET) method at *P* (equilibrium N_2_ pressure)/*P*_0_ (saturated N_2_ pressure) = 0.30 using a mixed gas of 30% N_2_–70% He (Flow Sorb II 2300, Shimadzu, Co., Kyoto, Japan). The sample was heated at 100 °C for 24 h to eliminate the adsorbed gas before BET measurement. The median size of alumina particles was measured by centrifugal sedimentation method of a dilute alumina suspension (CAPA-700; Horiba Ltd., Kyoto, Japan). The true density of α-alumina powder was measured with pycnometer using double-distilled water. The isoelectric point of alumina particles was measured in a 0.001 M NH_4_NO_3_ solution (Rank Mark II, Rank Brothers Ltd., Cambridge, UK). Figure 1 shows particle morphology of (a) rod-like particles, (b) disk-like particles, and (c) spherical particles. The length and width of rod-like particles were 200–400 nm and 100–200 nm, respectively. The diameter and thickness of disk-like particles were 1–10 μm and 1 μm, respectively. Although the median size measured by centrifugal sedimentation method was 1.47 μm, large particles above 10 μm were included in the disk-like particles. The spherical particles possessed a narrow particle size distribution of median size 0.60 μm.

### 2.2. Sintering of Porous Alumina Compacts

Colloidal processing was used to make alumina green compacts with uniform microstructures. The alumina particles in Table 1 were dispersed at a solid content of 10 vol% (rod-like particles and spherical particles) or 15 vol% (disk-like particles) in double-distilled water adjusted to pH 3 with a HNO_3_ solution. The positively charged alumina particles were stirred for 24 h at room temperature and then consolidated in a cylindrical vinyl chloride pipe mold (inner diameter of 15 mm, outer diameter of 18 mm, height of 30 mm) placed on a gypsum board for one week. The dried powder compacts were heated at 5 °C/min and sintered at 700–1600 °C in air for 1 h (SPM 6512 electric furnace, Marusho Denki Co. Ltd., Himeji, Japan). The sintered density and porosity were measured by the Archimedes method using double-distilled water. The specific surface area of sintered compact was measured by Brunauer–Emmett–Teller (BET) method (Flow Sorb II 2300, Shimadzu, Co., Kyoto, Japan). The microstructures of sintered alumina compacts were observed by field emission electron microscope (S-4100H FE-SEM, Hitachi High-Tech Technologies Co., Tokyo, Japan).

### 2.3. Young’s Modulus and Compressive Strength of Sintered Porous Alumina

The sintered alumina compact was cut into a cubic shape with 5 mm length. The alumina sample was sandwiched between two cupper plates (20 × 20 × 1 mm^3^). The sample was then compressed at a crosshead speed of 0.5 mm/min (Tensilon RTC, A&D Co. Ltd., Tokyo, Japan) while the strain along the compressive direction was measured using a strain gauge (4 mm × 3 mm) attached to the sample. The compressive test was performed with more than five samples for each sintering condition [15]. The Young’s modulus of the sintered compact was evaluated from the stress-strain curve up to 0.05% strain. The loading and unloading test was repeated more than five times for each sample. The sudden decrease of the compressive strain as shown in Section 3.3 reflected the compressive fracture.

## 3. Results and Discussion

### 3.1. Sintering Behavior

**Figure 2:**
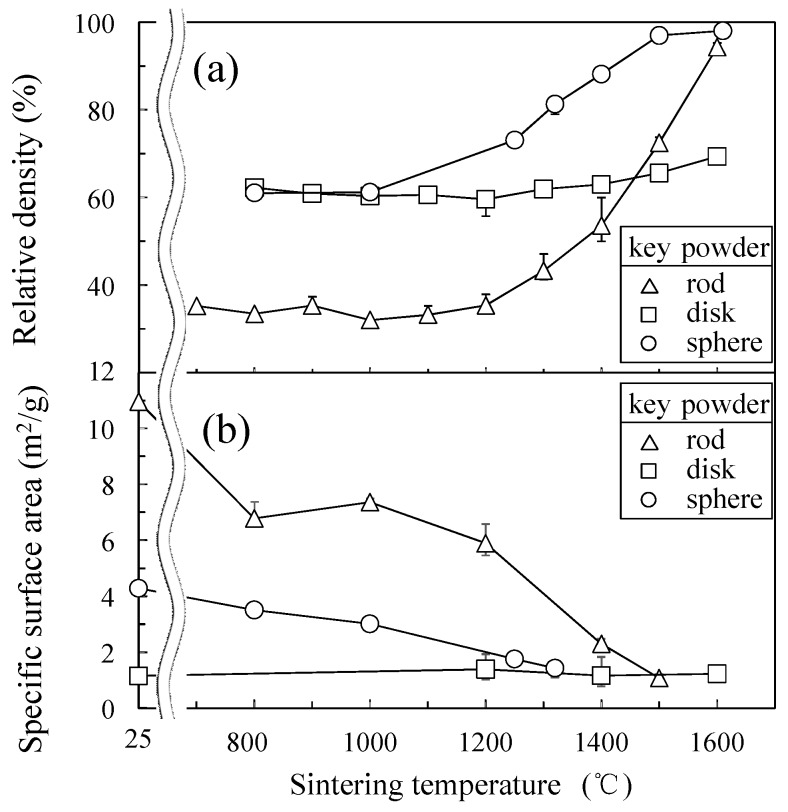
Relation between sintering temperature and (**a**) relative density or (**b**) specific surface area of sintered alumina compacts.

Figure 2 shows the relationship between sintering temperature and (a) relative density or (b) specific surface area of the sintered alumina compacts. The packing density of the rod-like particles (33.5%) was significantly low as compared with the disk-like particles (62.3%) or spherical particles (61.0%). However, the disk-like particles were hardly densified during the heating at 800–1600 °C. The sintering rates of the rod-like particles and spherical particles increased remarkably above 1200 °C and reached 94.3% and 98.0% of relative density at 1600 °C, respectively. The specific surface area of the disk-like particles did not change with sintering temperature, corresponding to little change of relative density. The specific surface areas of spherical particles and rod-like particles decreased gradually at a higher sintering temperature, reflecting the increase in the relative density with sintering temperature.

### 3.2. Microstructures of the Sintered Alumina Compacts

**Figure 3:**
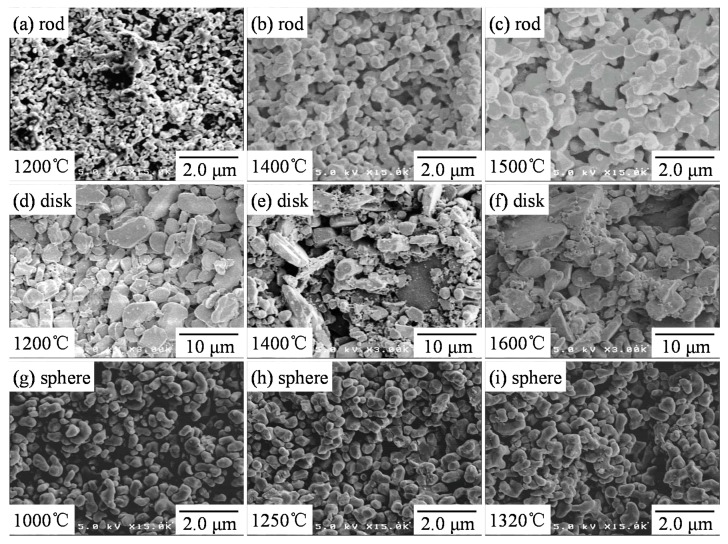
Microstructures of alumina compacts sintered from (**a**–**c**) rod-like particles, (**d**–**f**) disk-like particles, and (**g**–**i**) spherical particle at 1000–1600 °C.

Figure 3 shows the microstructures of alumina compacts sintered from a–c rod-like particles, d–f disk-like particles, and g–i spherical particles at 1000–1600 °C. The rod-like particles with a high specific surface area (10.96 m^2^/g, Table 1) and small sizes (0.55 μm median size, Figure 1) were densified with grain growth. The grain growth of primary rod-like particles occurred below 1200 °C without significant densification. The shape of rod-like particles changed to more spherical particles at a high sintering temperature. On the other hand, no significant change of the relative density and the microstructure was observed for the disk-like particles with a low specific surface area (1.16 m^2^/g) and the large particle sizes (1.47 μm median size). As compared with the rod-like particles or disk-like particles, the spherical particles were densified faster (Figure 2a) with little significant grain growth. As observed in Figure 2 and Figure 3, the particle shape provided great influence on the sintering behavior and grain growth rate.

### 3.3. Compressive Mechanical Properties

**Figure 4:**
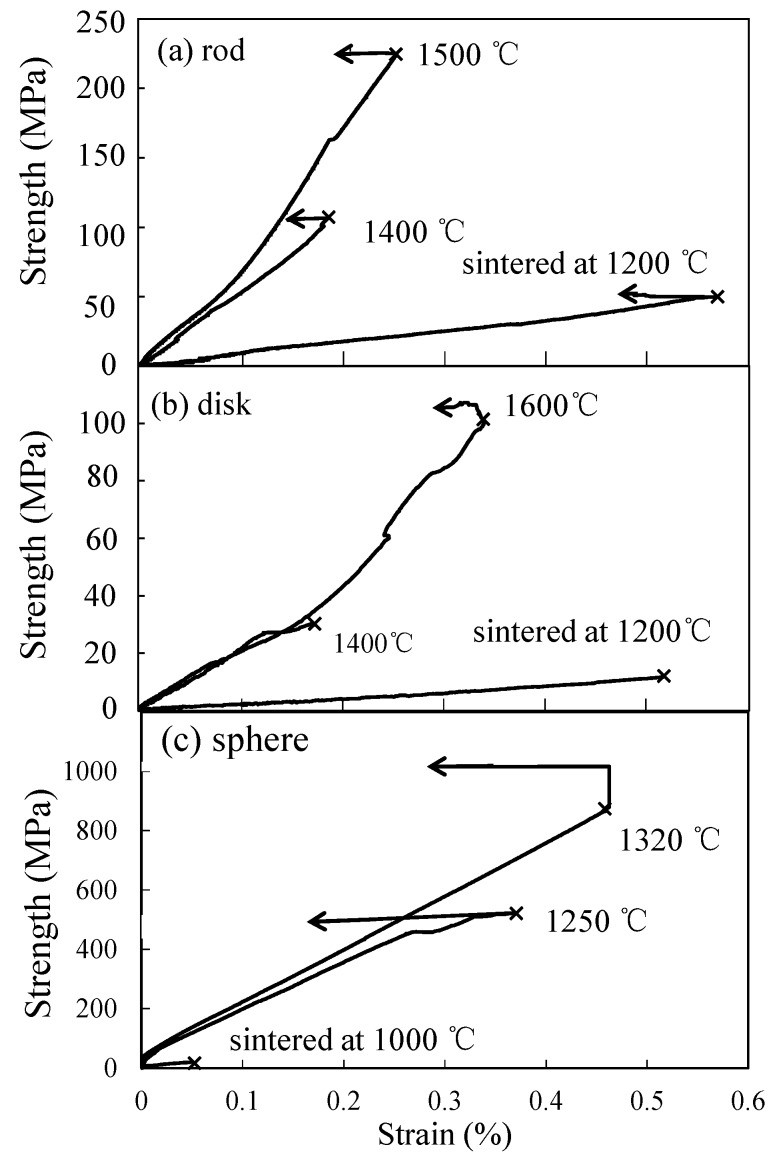
Compressive stress–strain curves for the alumina compacts sintered from (**a**) rod-like particles, (**b**) disk-like particles, and (**c**) spherical particles at 1000–1600 °C.

Figure 4 shows the compressive stress–strain curves for the alumina compacts sintered from (a) rod-like particles, (b) disk-like particles, and (c) spherical particles. The alumina compacts sintered at 1200 °C from the rod-like and disk-like particles showed the linear stress-strain relation but exhibited the nonlinear deformation curve with increasing sintering temperature. The compressive stress–strain curves for the alumina compacts from the spherical particles showed the high linearity of the stress-strain curves.

**Figure 5:**
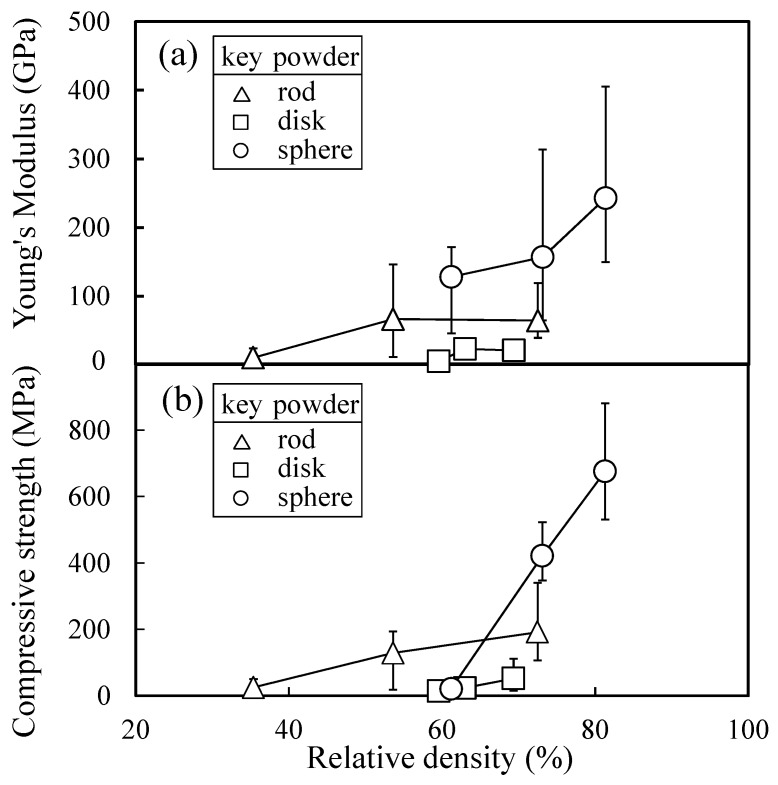
Relation between relative density and (**a**) Young’s modulus or (**b**) compressive strength.

Figure 5 summarizes the Young’s modulus and compressive strength as a function of relative density of sintered alumina compacts. The alumina compact from the disk-like particles showed the low Young’s modulus and low compressive strength, reflecting little formation of strong grain boundaries with heating. On the other hand, the Young’s modulus and compressive strength for the rod-like particles and spherical particles increased with increasing relative density. The increase in the relative density for these powders was accompanied by a decrease in the specific surface area of the alumina compacts, indicating the formation of strong grain boundaries as predicted by Equation (2).

**Figure 6:**
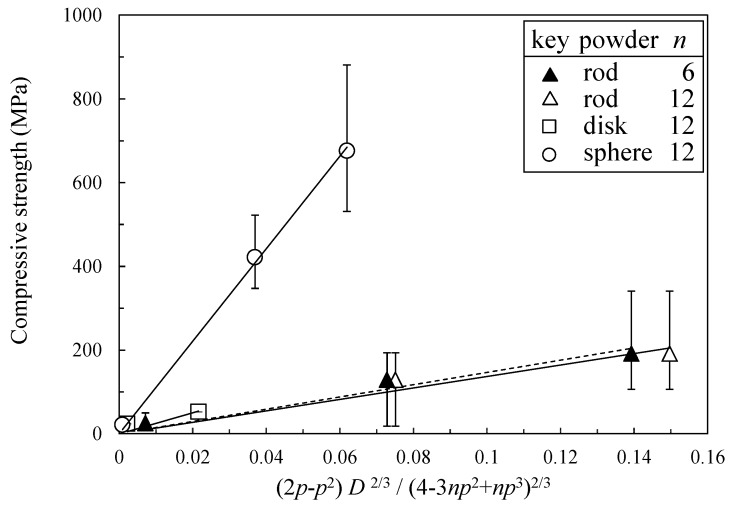
Relation between compressive strength and the right term of Equation (13) associated with grain boundary area.

Figure 6 shows the relationship between the compressive strength and the right term of Equation (13). The *p* values in Figure 6 were determined from Equations (8) and (9). The coordination number of *n* = 12 was applied for the disk-like particles and spherical particles because their packing densities of green compacts were close to the packing density of random closed packing of spherical particles (63.7%). In the case of disk-like particles, the coordination number of *n* = 12 or 6 was applied because of the low packing density of green compact. Although Equation (13) was derived for spherical particles, the same equation was also applied for the rod-like or disk-like particles to analyze the difference in the particle shape. In addition, the theoretical model explaining the mechanical properties for the different particle shape is not established at this moment. The relationship between the compressive strength and the right term function associated with the grain boundary area of Equation (13) for the three kinds of particles exhibited a good linearity. The compressive strength was higher for the spherical particles rather than for rod-like or disk-like particles. The scattering range of the Young’s modulus and strength in Figure 5 and Figure 6 is deeply related to the distribution range of the grain boundary areas developed in the open-pore structure. It was demonstrated in our paper [17] that the Young’s modulus was significantly sensitive to the porous microstructure.

### 3.4. Analysis of Compressive Strength

The compressive strength for fully dense alumina was calculated by Equations (8), (9), and (13) from the slope of the linear relationship in Figure 6, and expressed by Equation (14),

(14) $${\sigma (\mathrm{dense})=\sigma }_{0}F$$

where *F* corresponds to the *f*(*p*) value at *D* (relative density) = 1 in Equation (13). *F* values for rod-like, disk-like, and spherical particles were calculated as follows: *F* = 0.7197 for rod-like particles at *n* = 6, *D*_0_ = 0.335, *q* = 0.306, and *p* = 0.271, *F* = 0.8130 for rod-like particles at *n* = 12, *D*_0_ = 0.335, *q* = 0.306, and *p* = 0.244, *F* = 0.3488 for disk-like particles at *n* = 12, *D*_0_ = 0.623, *q* = 0.146, and *p* = 0.138, and *F* = 0.3639 for spherical particles at *n* = 12, *D*_0_ = 0.610, *q* = 0.152, and *p* = 0.143. The calculated *σ*(dense) value was 0.346 (*n* = 6)–0.366 GPa (*n* = 12) for the rod-like particles, 0.288 GPa for the disk-like particles (*n* = 12), and 1.320 GPa for the spherical particles (*n* = 12). The calculated *σ*(dense) value for the spherical particles was 40–60% of the reported compressive strength (2.2–3.3 GPa) of dense alumina ceramics [22,23], and was discussed in latter part of this section. However, the above calculated *σ*(dense) values should be close to each other at 0% porosity. The difference in the above *σ*(dense) values for different particle shape is interpreted as follows. The compressive strength of porous ceramics (*σ_p_*) is expressed by Equation (15) and related to the compressive strength of fully dense ceramics, *f*(*p*) value defined by Equation (13) (=3.046(2*p* − *p*^2^) *D*^2/3^/(4 − 3*np*^2^ + *np*^3^)^2/3^) and the number (*n_f_*) of grain boundaries which originally surrounded one grain and were fractured by a compressive stress.

(15) $$\sigma_{p}{=\sigma }_{0}f(p)=\frac{1}{2}n_{f}(\mathrm{porous})\sigma (\mathrm{dense})f(p)$$

In Equation (15), the *n_f_* value at fracture is distinguished from the coordination number (*n*) in *f*(*p*) value related to the sintering process. That is, the *σ*_0_ value in Equation (13) is expressed by Equation (16).

(16) $$\sigma_{0}(\mathrm{porous})=\frac{1}{2}n_{f}(\mathrm{porous})\sigma (\mathrm{dense})$$

For fully dense ceramics, Equation (17) is derived from Equation (14).

(17) $${\sigma (\mathrm{dense})=\sigma }_{0}(\mathrm{dense})F=\frac{1}{2}n_{f}(\mathrm{dense})\sigma (\mathrm{dense})F$$

In this experiment, *F* and *n_f_*(dense) values for the spherical particles were calculated to be 0.3639 and *n_f_* = 2/*F* = 5.50, respectively. The ratio of *n_f_* to *n* (coordination number = 12) resulted in 0.458. The ratio of *σ*_0_(porous)/*σ*_0_(dense) is calculated from Equations (16) and (17) as follows.

(18) $$\frac{\sigma_{0}(\mathrm{porous})}{\sigma_{0}(\mathrm{dense})}=\frac{n_{f}(\mathrm{porous})}{n_{f}(\mathrm{dense})}$$

When *σ*_0_ values for dense and porous compacts are measured, the ratio provides the *n_f_*(porous) value. Similarly, Equation (16) is useful to analyze the *n_f_*(porous) of partially sintered porous alumina for two kinds of particles as expressed by Equation (19).

(19) $$\frac{\sigma_{0}(\mathrm{porous},\;\mathrm{rod})}{\sigma_{0}(\mathrm{porous},\;\mathrm{spherical})}=\frac{n_{f}(\mathrm{porous},\;\mathrm{rod})}{n_{f}(\mathrm{porous},\;\mathrm{spherical})}$$

The measured *σ*_0_(porous, rod, *n* = 12)/*σ*_0_(porous, spherical, *n* = 12) resulted in 0.124, suggesting that the number of grain boundaries fractured by an applied stress was significantly smaller for rod-like particles or disk-like particles as compared with spherical particles. That is, the applied load concentrates on a few grain boundaries of rod-like particles, causing the fracture at a low compressive stress. The uniform distribution of the applied load over many grain boundaries of spherical particles contributes to the increase in the compressive strength of the partially sintered porous ceramics.

### 3.5. Observation of Fractured Surfaces

**Figure 7:**
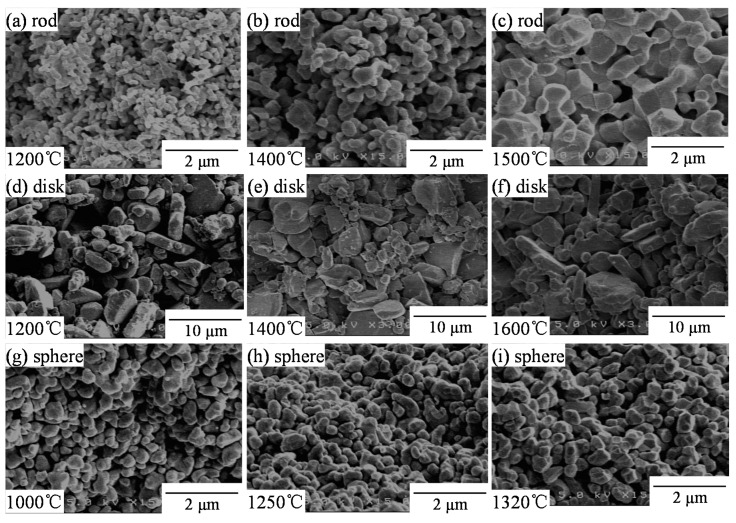
Fractured surfaces of alumina compacts sintered from (**a**–**c**) rod-like particles, (**d**–**f**) disk-like particles, and (**g**–**i**) spherical particle at 1000–1600 °C.

Figure 7 shows the fractured surfaces of alumina compacts sintered from a–c rod-like particles, d–f disk-like particles, and g–i spherical particles at 1000–1600 °C. The corresponding fractured strengths are shown in Figure 4. Those fractured microstructures were similar to the microstructures of as-sintered alumina compacts presented in Figure 3. Formation of polyhedral grains accompanied by the development of grain boundaries with increasing sintering temperature was clearly observed in the sintered alumina from rod-like particles (b,c) and spherical particles (h,i). From the fractured surfaces, it is apparent that the *n_f_*(porous) value is smaller than the coordination number (*n*) in the sintering process.

Since the microstructure (i) with small grains provided a higher compressive strength (average 676 MPa) than the microstructure (c) with large grains (average 192 MPa), the increase in *N* value (suppression of grain growth) in Equation (2) is important in addition to the large *n_f_* value in Equation (16) for the increased strength. The comparison of the microstructures between Figure 3f (sintered surface) and Figure 7f (fractured surface) for the disk-like particles suggests little formation of apparent grain boundaries, resulting in the low compressive strength. The above observation agreed basically with the analysis of strength in Section 3.4.

## 4. Conclusions

Three kinds of alumina particles with spherical, rod-like, and disk-like shape were examined for the sintering behavior and compressive strength of partially sintered porous alumina. The packing density of the rod-like particles was low but those particles were sintered densely with grain growth and with the shape change to more spherical particles. The disk-like particles showed a high packing density but no significant change was measured in the relative density and the microstructures with heating. The spherical particles exhibited a high packing density and were densified faster with little significant grain growth at a low heating temperature. The alumina compacts from the disk-like particles showed a low Young’s modulus and low compressive strength, reflecting little formation of strong grain boundaries with heating. On the other hand, the Young’s modulus and compressive strength for the rod-like particles and spherical particles increased with increasing relative density. The applied load concentrated on a few grain boundaries of disk-like or rod-like particles in the porous compacts, causing the fracture at a low compressive stress. The uniform distribution of the applied load over many grain boundaries of spherical particles contributed to the increase in the compressive strength of the partially sintered porous alumina. The above pore structure with high mechanical properties may be applied for the membrane filters used at a high pressure of gas or liquid, or for a porous electrode-supported solid oxide fuel cell.

## Figures and Tables

**Table 1:** Properties of starting alumina particles.

Powder No.	L30N2-F1112	ACLM-27	AKP20
Crystal structure	α-alumina	α-alumina	α-alumina
Manufacturer	Asahikasei	Sumitomo Chemical	Sumitomo Chemical
Particle shape	Rod-like	Disk-like	Spherical
Specific surface area (m^2^/g)	10.96	1.16	4.28
Median diameter (μm)	0.55	1.47	0.60
True density (g/cm^3^)	3.990	3.961	3.990
Isoelectric point	pH 6.45	pH 5.28	pH 5.31
